# Parental Teaching of Reading and Spelling Across the Transition From Kindergarten to Grade 1

**DOI:** 10.3389/fpsyg.2020.610870

**Published:** 2021-01-08

**Authors:** Gintautas Silinskas, Kaisa Aunola, Marja-Kristiina Lerkkanen, Saule Raiziene

**Affiliations:** ^1^Department of Psychology, University of Jyväskylä, Jyväskylä, Finland; ^2^Department of Teacher Education, University of Jyväskylä, Jyväskylä, Finland; ^3^Institute of Psychology, Vilnius University, Vilnius, Lithuania; ^4^Institute of Psychology, Mykolas Romeris University, Vilnius, Lithuania

**Keywords:** teaching of reading, teaching of spelling, home literacy, parents, reading, spelling

## Abstract

We investigated the longitudinal links between parental teaching of reading and spelling and children’s word reading and spelling skills. Data of 244 Lithuanian parent–child dyads were analyzed, who were followed across three time points: end of kindergarten (T1; *M*_*age*_ = 6.88; 116 girls), beginning of Grade 1 (T2), and end of Grade 1 (T3). The children’s word reading and spelling skills were tested, and the parents answered questionnaires on the frequency with which they taught their children reading and spelling. Overall, the results showed that the parents were responsive to their children’s skill levels across the domains of reading and spelling and across time (i.e., the transition from kindergarten to Grade 1 and across Grade 1). However, differences between the domains of reading and spelling were also observed. In particular, in the domain of reading and across the transition from kindergarten to Grade 1, the parents responded to their children’s skill levels by increasing the time spent teaching children with poor word reading skills, and decreasing the teaching time for the children with good word reading skills. In contrast, as spelling skills may require more time to develop, parents maintained similar frequencies of teaching spelling across the transition to Grade 1 for all children, and only parents of good spellers taught less spelling at the end of Grade 1 than parents of children with poor and average word spelling skills.

## Introduction

Early reading and spelling skills become relatively stable at the beginning of primary school and continue to improve thereafter (Lerkkanen et al., 2004a; Entwisle et al., 2005; Duncan et al., 2007; Torppa et al., 2017). Although schools are responsible for the development of children’s reading and spelling skills, parents and the home learning environments they create may be equally important in their development (Sénéchal and LeFevre, 2002, 2014; Manolitsis et al., 2013; Niklas and Schneider, 2013; Silinskas et al., 2020a,b). Although a plethora of studies on the effect of parental teaching on children’s skills exists, there are some limitations in the previous research that should be noted. First, previous studies have concentrated primarily on children’s reading development and the role of parents in facilitating it; far less is known about the development of children’s spelling skills and the parental role in this regard. Second, while most studies have assessed children’s skills, they have not followed parental responses simultaneously across time. To address this issue, longitudinal studies following parents across time are crucial. Relatedly, while the evidence from teaching in early years often shows positive associations with children’s literacy skills, less is known about these associations when children transition from kindergarten to Grade 1 and beyond. Finally, the relationships between parental home literacy activities and children’s reading and spelling skills have been examined extensively in North America, Western Europe, and Asia. However, to better understand the phenomenon of parental teaching and make broad generalizations, studies conducted in different cultural environments are needed. Consequently, the present study followed parental home literacy activities—the teaching of reading and spelling—from the end of kindergarten through to the end of Grade 1 with the goal of investigating their reciprocal longitudinal relations with children’s word reading and spelling skills. The data came from the unique cultural environment of Lithuania (the northeastern part of Europe), where formal literacy instruction begins in Grade 1 in the year of the child’s seventh birthday, and where the transparency of the Lithuanian language exposes children to quick reading acquisition but somewhat slower spelling acquisition (which is also typical in many other languages varying in orthographic consistency; Georgiou et al., 2020).

### Parental Teaching of Reading and Spelling in Kindergarten and Grade 1

As suggested by the Home Literacy Model (Sénéchal and LeFevre, 2002, 2014), a variety of literacy-promoting activities take place in children’s homes, including the parental instruction of formal literacy. Often labeled as mere teaching activities (Sénéchal and LeFevre, 2002, 2014), formal literacy activities define parental interaction with children regarding literacy when print *per se* is in focus. Typically, parents are mostly engaged in teaching the decoding-related aspects of literacy (e.g., letter names, sounds, or reading; Sénéchal and LeFevre, 2002, 2014). In particular, the teaching of reading before primary school has been found to be related to children’s early literacy skills, such as print concept awareness, letter knowledge, and decoding abilities (Sénéchal and LeFevre, 2002; Torppa et al., 2006). These associations have been demonstrated in opaque languages, such as English (Sénéchal and LeFevre, 2002; Hood et al., 2008; Stephenson et al., 2008) and French (Sénéchal, 2006), and similar results have been obtained in orthographically transparent languages, such as German (Lehrl et al., 2013; Niklas and Schneider, 2013, Niklas and Schneider, 2017), Greek (Manolitsis et al., 2011, 2013), and Finnish (Silinskas et al., 2020a,b).

The effects of home literacy activities have been well investigated; however, less is known about their spelling-related counterparts. This might be due to the fact that, in previous studies, the teaching of spelling and reading was often combined (Sénéchal, 2006; Niklas and Schneider, 2017). However, it has been shown that various spelling-related activities also take place in children’s homes (e.g., practicing how to spell one’s name; Aram and Levin, 2002; Levin and Aram, 2005; Aram et al., 2013). In the present study, we refer to these activities as the teaching of spelling. Although not explicitly examined as part of the Home Literacy Model (Sénéchal and LeFevre, 2002, 2014), spelling activities in kindergarten (or, to be precise, a combination of teaching reading and spelling) were shown to be related to the development of children’s spelling skills in kindergarten (Levy et al., 2006) and in Grades 1 and 4 (Sénéchal, 2006). Similar relationships were found in both opaque languages, such as English (Levy et al., 2006; Sénéchal, 2006), and transparent languages, such as German (Niklas and Schneider, 2013). In addition, a few observational studies on the quality of parental interaction during writing/word-printing tasks found a positive association with children’s word writing in kindergarten (Aram and Levin, 2002) and Grade 1 (Aram et al., 2013). Although these studies opened up an interesting direction for future research, they also placed emphasis on the quality, not frequency, of parental involvement and did not control for the effects of longitudinal autoregressors.

Research shows that the associations between the parental teaching of reading and spelling at home and children’s early reading and spelling skills vary depending on the children’s developmental stage (Bradley et al., 2001). That is, the associations are typically positive and stronger among younger children in comparison to primary school students. In studies conducted with kindergarteners, positive links have been found between the teaching of reading and early literacy skills at home (Sénéchal and LeFevre, 2002, 2014; Levy et al., 2006; Niklas and Schneider, 2015) and between the parental teaching of spelling and children’s spelling skills (Levy et al., 2006; Sénéchal, 2006). For children in Grades 1 and 2, some studies report that the teaching of reading in primary school has been positively related to reading (Sénéchal and LeFevre, 2014), whereas other studies have found no significant associations (Manolitsis et al., 2013) or negative ones (Silinskas et al., 2010, 2012). Research on the associations between the teaching of spelling and the development of spelling skills among primary school students is simply not available. Consequently, the examination of these links was among the aims of the present study.

### Reciprocity Between Reading and Spelling Skills and Parental Teaching

Traditionally, parental home activities have been assumed to enhance children’s early academic skills (Sénéchal and LeFevre, 2002, 2014; Hood et al., 2008; Stephenson et al., 2008); however, the opposite—when academic skills predict parental responses—is also true. These ideas are postulated by transactional theories of child socialization (Sameroff, 2010) and studies on the evocative effect of children’s characteristics on parental behaviors (Scarr and McCartney, 1983). Indeed, some research emphasizes that children’s characteristics, such as their motivation and skill levels, can evoke certain parental responses (Scarr and McCartney, 1983; Nurmi, 2012). Specifically, the evidence in such studies comes from school-aged children and shows that their academic performance may evoke parental responses that affect the frequency of teaching (Levin et al., 1997; Pomerantz and Eaton, 2001; Silinskas et al., 2012; Ciping et al., 2015). For instance, in examining the effects of Grade 2 students’ reading skills on parental activities, Sénéchal and LeFevre (2014) found that parents’ home literacy activities were influenced by the children’s previous word reading skills. In other words, parents whose children had stronger reading skills in Grade 1 reported fewer teaching activities in Grade 2; in contrast, parents whose children had poor reading skills in Grade 1 reported more teaching activities in Grade 2. Similar results have been obtained in the opaque language of Chinese (Ciping et al., 2015) and in highly transparent languages, such as Finnish (Silinskas et al., 2012), where it was found that the more frequent maternal teaching of reading at the end of Grade 1 could be predicted by lower reading skills in kindergarten and at the beginning of Grade 1.

The research on the reciprocity of home literacy activities and children’s emergent literacy skills comes primarily from the domain of reading, and much less is known about the domain of spelling. Moreover, the effect of children’s emergent spelling skills on parental teaching activities has not been examined by following the same children from kindergarten to Grade 1 (for exceptions in the domain of reading, see Silinskas et al., 2010, 2012; Sénéchal and LeFevre, 2014). While some cross-sectional evidence does exist, longitudinal cross-lagged analyses of parental activities and skills across time must be performed to investigate the reciprocal associations between parental teaching activities at home and children’s literacy skills. The associations may be different for reading and spelling models because spelling skills have been shown to develop more slowly than reading skills (Lerkkanen et al., 2004b; Leppänen et al., 2006; Landerl and Wimmer, 2008; Hirvonen et al., 2010; Georgiou et al., 2020). Thus, parents may adapt their teaching of spelling later in school, whereas they may be more reactive to children’s reading skills earlier on at the start of Grade 1.

The research carried out thus far has explained the negative associations between parental teaching and early reading skills by assuming that parents increase their frequency of teaching in response to children’s poor reading skills and comparisons with the parents of children with better skills. However, the alternative explanation for the negative associations can also be true: It is possible that the parents of good readers or spellers decrease their frequency of teaching in comparison to the parents of children with poorer skills. Additionally, both explanations could be simultaneously true; however, these alternative possibilities have not been investigated in previous research. Therefore, in addition to the associations between the variables, the investigation of the mean-level differences will further advance our understanding of the phenomenon.

### Reading and Spelling in the Lithuanian Educational System

Lithuanian children enter kindergarten on the first of September of the calendar year of their sixth birthday. In Lithuania, kindergarten education takes place 1 year before Grade 1 and became compulsory in 2016 (LR Ministry of Education, Science and Sports, 2014a). The aim of kindergarten education in Lithuania is to ensure the optimal development of the child’s individual qualities and to prepare him or her to learn according to the primary-education curriculum (LR Ministry of Education, Science and Sports, 2014a). Kindergarten education strategies are child-centered, and teachers’ practices must accord with the kindergarten education curriculum, which is divided into five domains (competencies): social, health, cognitive, communication, and art. Only a small portion of these competences concern the development of children’s reading and spelling skills *per se* (e.g., recognizing similarities and differences of sounds, connecting sounds and letters, recognizing and writing letters, reading individual words, differentiating between uppercase and lowercase letters, and practicing spelling using capital letters; LR Ministry of Education, Science and Sports, 2014a). However, kindergarten teachers have significant autonomy in choosing their pedagogical practices, which are confirmed by the school, and considering the individual needs of the children (LR Ministry of Education, Science and Sports, 2014b). Moreover, the kindergarten curriculum does not set criteria for determining the levels of reading and spelling skills before school entrance. It is only in Grade 1 that children are exposed to the systematic teaching/learning of reading and spelling at school.

### Research Questions

The goal of the present study was to examine the longitudinal reciprocal links between the frequency of parental teaching of reading and spelling and the development of children’s word reading and spelling skills across the transition from kindergarten to the beginning and end of Grade 1. The following research questions (RQs) were thus examined:

(RQ 1a) To what extent does the frequency of parental teaching of reading predict children’s subsequent word reading skills?

(RQ 1b) To what extent does the frequency of parental teaching of spelling predict subsequent word spelling skills?

As shown previously, we expected the associations between parental activities and children’s literacy skills in the kindergarten–Grade 1 transition to be positive (e.g., Sénéchal and LeFevre, 2002; Hood et al., 2008; Manolitsis et al., 2011, 2013; Niklas and Schneider, 2013, 2017). The previous literature has provided mixed results suggesting positive, zero, or negative associations between teaching of reading and reading skills in Grade 1 (Silinskas et al., 2010, 2012; Sénéchal and LeFevre, 2014). Consequently, we set no specific hypotheses for Grade 1.

(RQ 2a) To what extent do children’s word reading skills predict the frequency of the parental teaching of reading?

(RQ 2b) To what extent do children’s spelling skills predict the frequency of the subsequent parental teaching of spelling?

In keeping with previous research in the domain of reading during the kindergarten–Grade 1 transition (Silinskas et al., 2010, 2012) and in the early grades of primary school (Sénéchal and LeFevre, 2014), we expected negative paths between children’s word reading skills and the subsequent frequency of parental teaching activities. Due to the lack of previous evidence, we did not set any specific expectations for the domain of spelling.

Previous research has shown that certain child and parent characteristics—that is, child gender and parental education—are associated with children’s skills and parental teaching patterns (Davis-Kean, 2005; Silinskas et al., 2020b); therefore, we controlled for their effects in the present longitudinal investigation. In addition, we controlled for parental beliefs regarding the importance of their children reaching certain skill levels in reading and spelling before they entered Grade 1 (Aunola et al., 2002; Sénéchal and LeFevre, 2002; Martini and Sénéchal, 2012; Kluczniok et al., 2013; Skwarchuk et al., 2014).

## Materials and Methods

### Participants and Procedure

The present study is a part of the longitudinal data collection project “Get involved!” (Silinskas and Raiziene, 2017–2018), which followed Lithuanian children and their parents across the transition from kindergarten to Grade 1. The study protocol was approved by the Ethical Committee of the University of Jyväskylä (3.5.2017). We initially approached six principals, who granted us permission to collect data at their schools. The three smaller schools were located in the rural/provincial parts of the country (35% of the study participants), while the other three were located in Vilnius, the largest city and the capital of Lithuania (65% of the study participants). In this way, the proportion of our sample represents the proportion of the children attending kindergartens in Lithuanian provinces and cities (35 and 65%, respectively).

All participating kindergarten classes were situated in the same buildings as the children’s future primary schools, and all the schools were Lithuanian-speaking. Regarding their home language environments, 89.8% of the children spoke only Lithuanian at home, and 7.3% spoke a combination of Lithuanian and Russian or Polish. In particular, 2.1% spoke only Russian at home, and 0.8% spoke only Polish at home, but these children did not differ from the rest in terms of any of the study variables. This language profile of our sample is quite representative of the overall population of Lithuania, where the minority languages most commonly spoken at home are Polish (6%) and Russian (5%) (Statistics Lithuania, 2014). Moreover, the sample was highly homogeneous in regard to the ethnic and cultural backgrounds of the study participants, which is typical of the school population in Lithuania. In terms of parental education (Statistics Lithuania, 2014), our sample comprised somewhat highly educated mothers: 61.7% reported that they had obtained a university degree, 23.8% had completed a college or polytechnic program, 9.7% had completed high school, and only 4.8% had completed a level lower than high school.

#### Children

The children were tested individually to assess reading fluency and spelling skills on three occasions: the end of kindergarten (T1; April–May, 2017; *n* = 244; 127 girls), the beginning of Grade 1 (T2; October–November, 2017; *n* = 184), and the end of Grade 1 (T3; April–May; *n* = 186). On each occasion, testing took place in the office of the school psychologist, who administered the test. On the first testing occasion, many of the children were approaching 7 years of age (*M_age_* = 6.79, *SD* = 0.47). A total of 87.2% of the participants had attended preschool prior to the compulsory kindergarten year.

A total of 44 children dropped out of the study between T1 and T2 because they moved away, changed schools, or were not present at the schools for the testing periods. The sample size increased again between T2 and T3 by two children. The analyses of the missing data revealed no systematic differences between the children who had dropped out, stayed in the study, or joined the study at any time point.

#### Parents

A total of 244 of the parents filled in questionnaires in the spring of the kindergarten year (T1), 187 at the beginning of Grade 1 (T2), and 180 at the end of Grade 1 (T3). The questionnaires were completed by mothers (92.2%), fathers (4.9%), both parents together (1.6%), or other guardians (1.2%; e.g., grandmother or foster-care professional). The ages of the parents/guardians ranged from 23 to 60 (*M* = 35.40, *SD* = 5.46). Regarding family structure, 79.1% of the children were from two-parent families, 4.5% were from families in which the mother or father lived with his or her new spouse and their children, 11.5% lived with a single mother or father, and 4.9% of the guardians reported “other” (e.g., grandparents or foster-care professionals).

A total of 57 parents dropped out of the study between T1 and T2 because their children had started attending other schools. Between T2 and T3, seven more parents dropped out of the study. At T1 and T2, the sample size of parents was higher than that of children; this is because not all children whose parents filled out questionnaires were tested (e.g., due to some classrooms being quarantined or to the absence of parental consent to test the children even if the same parents answered the questionnaires). The analyses of the missing data revealed no systematic differences between the parents who dropped out at any point in time and those whose data were available.

### Measures

All the measures used in this study were developed based on those employed in the Finnish First Steps longitudinal study (Lerkkanen et al., 2006–2016) and another longitudinal study in Lithuania (Gedutiene, 2008). We also considered the guidelines of the Lithuanian Kindergarten Curriculum (LR Ministry of Education, Science and Sports, 2014a) when developing the measures for early reading and spelling skills and the parental teaching activities in relation to these skills. The psychometric properties of all study variables are presented in Table 1.

**TABLE 1:** Psychometric properties of all study variables.

				**Reliability**	**Range**		
	***n***	***M***	***SD***	**(Cronbach’s α)**	**Potential**	**Actual**	**Skewness**
**Teaching of reading**							
End of kindergarten (T1)	244	2.95	1.00	0.73	1–5	1–5	0.03
Beginning of Grade 1 (T2)	187	2.89	1.23	0.81	1–5	1–5	–0.03
End of Grade 1 (T3)	180	2.41	1.15	0.83	1–5	1–5	0.64
**Teaching of spelling**							
End of kindergarten (T1)	244	2.72	1.02	0.73	1–5	1–5	0.37
Beginning of Grade 1 (T2)	187	2.85	1.12	0.81	1–5	1–5	0.17
End of Grade 1 (T3)	180	2.19	1.16	0.83	1–5	1–5	1.00
**Word reading**							
End of kindergarten (T1)	228	6.69	5.94	0.96^*a*^	0–16	0–16	0.32
Beginning of Grade 1 (T2)	184	15.57	11.50	0.96^*a*^	0–75	0–57	1.01
End of Grade 1 (T3)	186	24.48	12.42	0.97^*a*^	0–75	0–61	0.46
**Word spelling**							
End of kindergarten (T1)	228	18.61	10.24	0.97	0–32	0–32	–0.53
Beginning of Grade 1 (T2)	184	27.52	10.49	0.91	0–40	0–40	–1.26
End of Grade 1 (T3)	186	35.21	5.74	0.94	0–40	2–40	–3.33
**Control variables**							
Child’s gender (0 girl; 1 boy)	244	0.48	0.50		0–1	0–1	0.08
Maternal education ^*b*^	227	4.42	0.87		1–5	1–5	–1.49
Belief: Benchmark for reading	243	4.32	0.80	0.86	1–5	1–5	–1.19
Belief: Benchmark for spelling	243	4.44	0.74	0.87	1–5	1–5	–1.62

*^*a*^The Kuder-Richardson reliability, a measure of internal consistency for dichotomous variables. ^*b*^Maternal education was assessed on a 5-point scale: lower than middle school, middle school, high school, college or polytechnics, and university degree.*

#### Parent Questionnaire (T1, T2, and T3)

##### Teaching of reading

Based on the dissertation of Gedutiene (2008) and the First Steps study by Lerkkanen et al. (2006–2016), three questions were developed to measure the parental teaching of reading. These three questions were used at all three measurement points. The parents were asked the following: *How frequently have you and your child been learning/practicing (1) to recognize letters, (2) to recognize sounds, and (3) to read words?* The question targeted both the current and retrospective frequencies of the parental teaching activities, including those *during this school year (from September)* for T1 and T2 and those *since Christmas* for T3. The parents used a six-point scale to answer the questions (0 = *not anymore, because my child has mastered the skill*, 1 = *never*, 2 = *rarely*, 3 = *sometimes*, 4 = *often*, and 5 = *very often*). If parents stated that they no longer engaged in these activities because their children had mastered the skill, this response was coded with a value of 1, thus making the scale range from 1 to 5. Cronbach’s alphas for the scale were 0.73, 0.81, and 0.83 for T1, T2, and T3, respectively.

##### Teaching of spelling

Based on the work of Sénéchal (2006) and Gedutiene (2008), three questions were developed to measure the parental teaching of spelling. These three questions were also used at all three measurement points. The parents were asked the following: *How frequently have you and your child been learning/practicing to (1) write letters, (2) write the child’s name, and (3) spell words?* The questions targeted both the current and retrospective frequencies of the parental teaching activities, including those *during this school year (from September)* for T1 and T2 and those *since Christmas* for T3. The parents used a six-point scale to answer the questions (0 = *not anymore, because my child has mastered the skill*, 1 = *never*, 2 = *rarely*, 3 = *sometimes*, 4 = *often*, and 5 = *very often*). If the parents stated that they no longer engaged in these activities because their children had mastered this skill, the response was coded with a value of 1, thus making the scale range from 1 to 5. Cronbach’s alphas for the scale were 0.73, 0.81, and 0.83 for T1, T2, and T3, respectively.

##### Beliefs concerning the benchmarks that a child needs to achieve before entering Grade 1 (measured at T1 only)

Based on the work of Martini and Sénéchal (2012) and Kluczniok et al. (2013), these beliefs were measured on a five-point scale (1 = *not really important*, 2 = *important to some extent*, 3 = *quite important*, 4 = *important*, and 5 = *very important*). The overall question was as follows: *How important do you consider the following skills and their teaching in preschool education to be?* For the **benchmark for reading**, the parents rated the importance of developing three skills: *(1) recognizing letters, (2) recognizing sounds*, and *(3) reading a few words*. For the **benchmark for spelling**, we asked about three skills: *(1) writing letters, (2) writing the child’s name*, and *(3) spelling a few words*. Cronbach’s alphas were 0.859 and 0.867 for the parental beliefs about benchmarks in reading and spelling, respectively.

#### Child Tests (T1, T2, and T3)

##### Word reading

An individually administered reading fluency test based on the Lukilasse test (6- to 12-year-old children; Häyrinen et al., 1999) and the work of Gedutiene (2008) was used. At the end of kindergarten (T1), the child was presented with a list of 16 real words, and in Grade 1 (T2 and T3), the child was presented with 75 real words divided into three columns. The words ranged from one to four syllables and were written in uppercase letters. The child was instructed to read the words aloud, and the score was based on the number of words read correctly within a 45-s time frame.

##### Word spelling

An individually administered spelling test based on the work of Gedutiene (2008) was used. At the end of kindergarten (T1), the child was presented with a list of eight real words, and in Grade 1 (T2 and T3), the child was presented with 10 real words. The words, which were organized in order of difficulty, ranged from two to three syllables and four to 11 letters in length. The child was asked to spell the words to the best of his or her ability as the tester read the words aloud one at a time. The child had as much time as necessary to write each word. All written words were scored from 0 to 4 (0 = incorrectly spelled word; 0.5 = one correctly spelled letter, but not the first letter; 1 = only the first letter of the word spelled correctly; 2 = two or more correctly spelled letters; 3 = the word is spelled incorrectly, but contains the correct phonetic structure and/or switched letters; 4 = correctly spelled word).

#### Analysis Strategy

Structural equation modeling was carried out using the M*plus* statistical package (version 8.4; Muthén and Muthén, 1998–2017). The analysis of the missing data was performed using Little’s (1988) missing completely at random (*MCAR*) test. Little’s MCAR test supported the null hypothesis (χ^2^ [140] = 138.968, *p* = 0.509), suggesting that the data were MCAR. As a result, the models were estimated using full information maximum likelihood estimation with robust standard errors (FIML), which is considered robust to non-normality and uses all available information to estimate the model (Muthén and Muthén, 1998–2017). Model fit was examined using a combination of chi-square (χ^2^), the Tucker-Lewis index (TLI), the comparative fit index (CFI), the root mean square error of approximation (RMSEA), and the standardized root mean square residual (SRMR). Non-significant χ^2^, TLI, and CFI values above 0.95; RMSEA value below 0.06; and SRMR value below 0.08 indicated a good model fit (Hu and Bentler, 1999). TLI and CFI values above 0.90, and RMSEA and SRMR values below 0.10 indicated an acceptable fit (Kline, 2016). We also calculated 95% confidence intervals for RMSEA. If the confidence interval did not span zero, then it indicated that the model fit was good.

To answer our research questions, we applied random intercept cross-lagged panel models (RI-CLPM; Hamaker et al., 2015). Our modeling strategy was selected based on the concern that the traditional cross-lagged models do not yield interpretable estimates due to the mixing of between-person and within-person variances (Curran et al., 2014; Hamaker et al., 2015; Berry and Willoughby, 2017). As suggested by Hamaker et al. (2015), latent variables at the within-person level represent the within-person changes around the overall level of the individual, and the latent constructs at the between-person level represent the stable interindividual differences over the whole assessment period. We built two models: one for reading and one for spelling. When building our RI-CLPM models for reading and spelling, we used the mean scores of parental teaching activities and children’s skills; thus, we had only one indicator per construct. Before proceeding with the model specifications, all variables were standardized.

We started by estimating four separate RI-CLPM models: the teaching of reading, word reading fluency, the teaching of spelling, and word spelling skills. Then, the separate skill and teaching models were combined to form our two final models: the reading model and the spelling model. In these two models, all stability and cross-lagged paths were included. In addition, the correlations between the T1 measures and the covariances between the unexplained variances of the within-person factors within each subsequent time point (T2 and T3) were estimated. The between-level factors were specified to be correlated with each other but not with any of the within-person factors. As the final step, to control for the possible impacts of gender, maternal education, and parental beliefs (i.e., benchmarks for reading and spelling before Grade 1) on the cross-lagged relations between teaching and skills, these control variables were included in the final model by estimating the paths from the variables to all within-level factors.

## Results

### Descriptive Analyses

The descriptives of all the study variables are presented in Table 1. Repeated measures ANOVAs revealed a significant decrease across time in the teaching of reading (*F*[2 348] = 19.10, *p* < 0.001, partial η^2^ = 0.10) and the teaching of spelling (*F*[2 348] = 35.48, *p* < 0.001, partial η^2^ = 0.17). Similarly, a significant increase in word reading fluency (*F*[2 354] = 494.24, *p* < 0.001, partial η^2^ = 0.74) and word spelling skills across time (*F*[2 354] = 511.47, *p* < 0.001, partial η^2^ = 0.74) was found. The correlations among all study variables are presented in Table 2. They show medium stability for the teaching of reading (0.306–0.671) and the teaching of spelling (0.397–0.626) across time, and links between the teaching of reading and the teaching of spelling in T1 (0.707), T2 (0.755) and T3 (0.846). The correlations between the teaching behaviors and skills were negative, ranging from –0.165 to –0.641 in the domain of reading, and from –0.122 to –0.405 in the domain of spelling.

**TABLE 2:** Correlations between all study variables.

	**Teaching of reading**			**Teaching of spelling**			**Word reading**			**Word spelling**			**Control variables**		
	**1.**	**2.**	**3.**	**4.**	**5.**	**6.**	**7.**	**8.**	**9.**	**10.**	**11.**	**12.**	**13.**	**14.**	**15.**
**Teaching of reading**															
1. End of kindergarten (T1)	1														
2. Beginning of Grade 1 (T2)	0.356**	1													
3. End of Grade 1 (T3)	0.306**	0.671**	1												
**Teaching of spelling**															
4. End of kindergarten (T1)	0.707**	0.353**	0.401**	1											
5. Beginning of Grade 1 (T2)	0.357**	0.755**	0.623**	0.472**	1										
6. End of Grade 1 (T3)	0.249**	0.561**	0.846**	0.397**	0.626**	1									
**Word reading**															
7. End of kindergarten (T1)	−0.165*	−0.641**	−0.568**	−0.185**	−0.389**	−0.451**	1								
8. Beginning of Grade 1 (T2)	−0.237**	−0.604**	−0.538**	−0.262**	−0.382**	−0.408**	0.838**	1							
9. End of Grade 1 (T3)	−0.220**	−0.523**	−0.499**	−0.278**	−0.342**	−0.348**	0.723**	0.872**	1						
**Word spelling**															
10. End of kindergarten (T1)	–0.107	−0.545**	−0.495**	−0.140*	−0.363**	−0.405**	0.821**	0.730**	0.638**	1					
11. Beginning of Grade 1 (T2)	–0.061	−0.475**	−0.509**	–0.122	−0.277**	−0.386**	0.697**	0.704**	0.660**	0.854**	1				
12. End of Grade 1 (T3)	–0.094	−0.337**	−0.396**	−0.156*	−0.223**	−0.318**	0.473**	0.503**	0.585**	0.603**	0.740**	1			
**Control variables**															
13. Child’s gender (0 = girl; 1 = boy)	–0.019	0.144*	0.176*	0.033	0.107	0.151*	−0.187**	−0.195**	–0.118	−0.197**	−0.245**	−0.159*	1		
14. Maternal education	−0.183**	−0.213**	−0.255**	−0.219**	−0.307**	−0.274**	0.273**	0.288**	0.324**	0.297**	0.304**	0.394**	–0.012	1	
15. Belief: Benchmark for reading	0.137*	–0.052	–0.023	0.166**	–0.022	–0.017	0.186**	0.110	0.051	0.174**	0.160*	0.015	–0.077	0.076	1
16. Belief: Benchmark for spelling	0.095	–0.016	0.024	0.159*	–0.005	0.040	0.118	0.077	0.018	0.108	0.110	0.036	–0.068	0.015	0.842**

*p < 0.05, **p < 0.01.

### Longitudinal RI-CPLM Models for Parental Teaching and Child Skills

#### Reading Model

**FIGURE 1:**
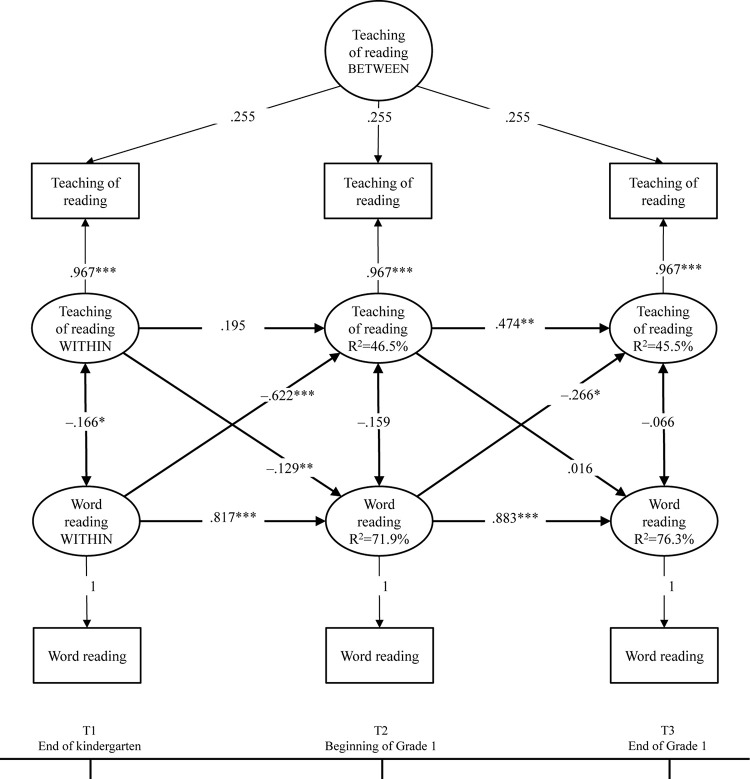
Parental teaching of reading and children’s word reading fluency across kindergarten and Grade 1. Standardized solution of the RI-CLPM. ^∗^*p* < 0.05, ^∗∗^*p* < 0.01, ^∗∗∗^*p* < 0.001.

The initial results revealed that the between-level factor for word reading fluency had a negative variance, indicating that there was no individual variation in the stable part of word reading fluency. Consequently, the final model included the between-level factor for only the teaching of reading. The model had a good fit (χ^2^ [3] = 3.349, *p* = 0.322; TLI = 0.995; CFI = 0.999; RMSEA = 0.026, 90%CI[0.001–0.014]; and SRMR = 0.010). The results are presented in Figure 1. The within-person factors represented the individual fluctuation around their overall level, denoted as WITHIN (in ovals, other than between). The positive autoregressive effects suggested that fluctuation from the overall level was predicted by a similar difference from the overall level at a previous time point. However, this was not significant for the teaching of reading across T1–T2. The within-person factors of the teaching of reading and word reading fluency correlated negatively at the end of kindergarten (T1). Most importantly, the cross-lagged associations across time suggested that word reading fluency at T1 and T2 negatively predicted the subsequent teaching of reading. In particular, word reading fluency in kindergarten negatively predicted the parental teaching of reading at the beginning of Grade 1 (β = –0.622, *p* < 0.001), and word reading fluency at the beginning of Grade 1 negatively predicted the parental teaching of reading at the end of Grade 1 (β = –0.266, *p* = 0.012). Additionally, the teaching of reading at the end of kindergarten (T1) negatively predicted word reading fluency at the start of Grade 1 (T2) (β = –0.129, *p* = 0.012).

#### Spelling Model

**FIGURE 2:**
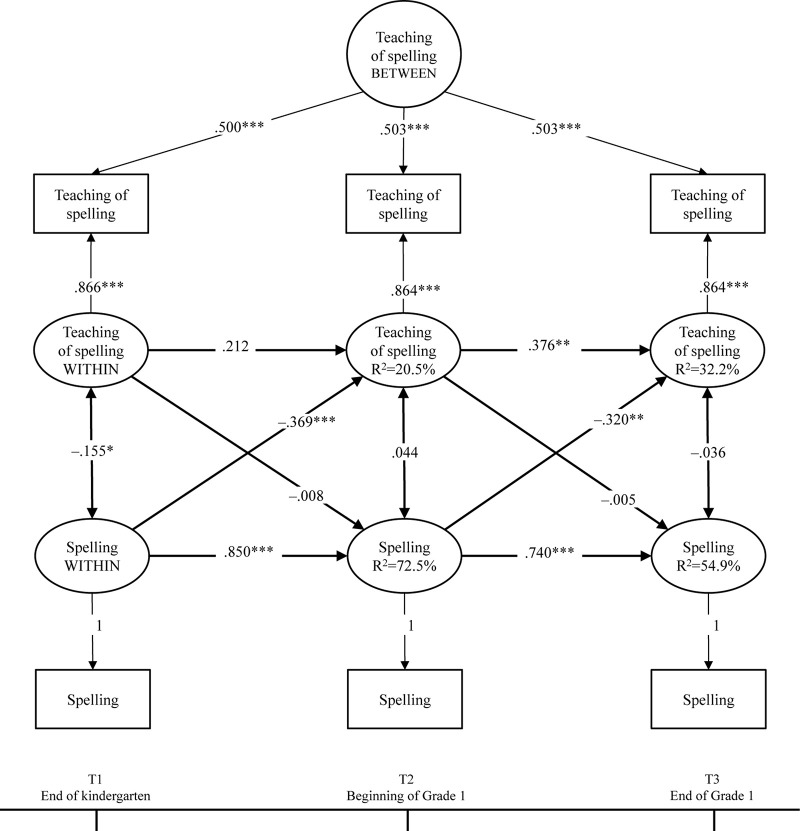
Parental teaching of spelling and children’s word spelling skills across kindergarten and Grade 1. Standardized solution of the RI-CLPM. ^∗^*p* < 0.05, ^∗∗^*p* < 0.01, ^∗∗∗^*p* < 0.001.

An identical procedure was repeated with the spelling model. The initial results revealed that the between-level factor of spelling skills had a negative variance, indicating that there were no individual variations in the stable part of spelling skills. Consequently, the final model included a between-level factor for only the teaching of spelling. The model had a good fit (χ^2^ (3) = 2.643, *p* = 0.450; TLI = 1.000; CFI = 1.004; RMSEA = 0.001, 90%CI[0.001–0.103]; and SRMR = 0.012). The results are presented in Figure 2. The positive autoregressive effects suggested that fluctuation from the overall level was predicted by a similar difference from the overall level at a previous time point. However, once again, this was not significant for the teaching of spelling across T1–T2. The within-person factors of the teaching of spelling and spelling correlated negatively at the end of kindergarten (T1). Most importantly, the cross-lagged associations across time suggested that spelling skills at T1 and T2 negatively predicted the subsequent parental teaching of spelling; that is, spelling skills in kindergarten negatively predicted the parental teaching of spelling at the beginning of Grade 1 (β = –0.369, *p* < 0.001), and spelling skills at the beginning of Grade 1 negatively predicted the parental teaching of spelling at the end of Grade 1 (β = –0.320, *p* = 0.001).

### Roles of Child Gender, Maternal Education, and Parental Beliefs

To ensure that the results concerning cross-lagged effects between teaching and word reading/spelling skills were not dependent on child gender, maternal education, and parental beliefs, these control variables were added to the model to predict all the within-level factors. For both the reading and spelling models, after controlling for these variables, the results concerning the reciprocal effects remained the same as previously reported. Furthermore, in the reading model, the results for the control variables showed that girls read better than boys at the end of kindergarten (T1) (β = –0.168, *p* = 0.007). Maternal education predicted less teaching of reading at T1 (β = –0.199, *p* = 0.002) and higher word reading performance at T1 (β = 0.271, *p* < 0.001), T2 (β = 0.079, *p* = 0.017), and T3 (β = 0.080, *p* = 0.036). Parental beliefs about the children’s benchmarks in reading positively predicted more frequent parental teaching at the end of kindergarten (T1) (β = 0.152, *p* = 0.022) and significantly predicted a higher level of word reading fluency at T1 (β = 0.146, *p* = 0.019). No other paths were significant. After identical model specifications in the spelling model, the findings showed that girls were more likely to have better spelling skills than boys at the end of kindergarten, or T1 (β = –0.190, *p* = 0.002). Maternal education predicted less teaching of spelling at T1 (β = –0.257, *p* = 0.002) and T2 (β = –0.217, *p* = 0.022) and higher spelling performance at T1 (β = 0.303, *p* < 0.001), T2 (β = 0.091, *p* = 0.049), and T3 (β = 0.221, *p* = 0.003). Parental beliefs about the children’s benchmarks in spelling predicted more parental teaching of spelling at the end of kindergarten (T1) (β = 0.189, *p* = 0.005). No other paths were significant.

### Mean-Level Differences in Teaching Activities Across Time: Moderation of Children’s Skill Levels

To better understand our correlational results, we took an approach similar to the one in previous analyses in the domain of reading (Sénéchal and LeFevre, 2014; Silinskas et al., 2020a) and continued investigating the mean-level differences between the teaching of reading and spelling activities for groups of children with different levels of skills. Participants with missing values for either teaching activities at any time point or skills at T3 were excluded from the analyses. The remaining sample (*n* = 175) was divided into three groups based on the children’s word reading fluency and spelling skills at the end of Grade 1 (T3). The word reading fluency or spelling skill groups were labeled as poor (< 25th percentile; *n* = 42 for reading and *n* = 46 for spelling), average (25th to 75th percentile; *n* = 91 for reading and *n* = 91 for spelling), and good (> 75th percentile; *n* = 42 for reading and *n* = 38 for spelling). Subsequently, we analyzed the progression of the mean scores for the parent-reported frequencies of teaching activities in a Time × Skill mixed ANOVA. In the analyses, Time referred to T1, T2, and T3 as a within-subject variable, and Skill (poor, average, or good) was a between-subject variable.

**FIGURE 3:**
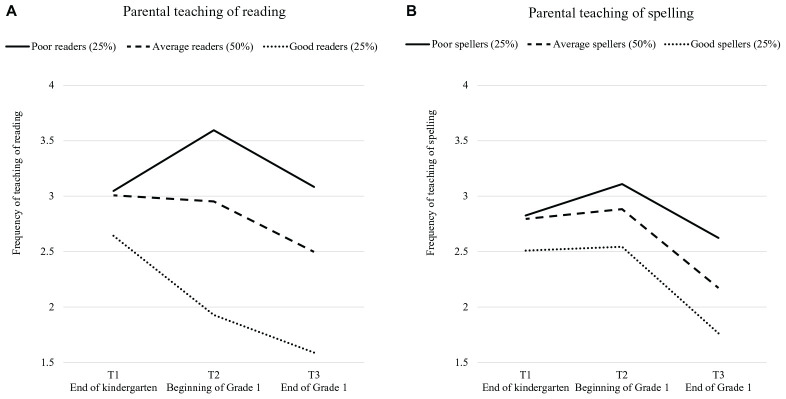
Mean-level differences in the frequency of parental teaching of reading **(A)** and spelling **(B)** across time for children with different skill levels.

In the analyses of word reading fluency (Figure 3A), we found significant main effects of Time (*F*[2, 344] = 17.85, *p* < 0.001, η_*p*_^2^ = 0.09) and Skill (*F*[2, 172] = 23.59, *p* < 0.001, η_*p*_^2^ = 0.22). The main effects were qualified by a significant Time × Skill interaction (*F*[4, 344] = 7.56, *p* < 0.001, η_*p*_^2^ = 0.08). *Post hoc* Bonferroni contrasts were then calculated. On average, there was no mean difference in the teaching of reading in T1 and T2, whereas the mean-level teaching of reading at T3 was lower than the means at T1 (Δ*M* = –0.509, *SE* = 0.099, *p* < 0.001; Cohen’s *d* = 0.471) and T2 (Δ*M* = –0.435, *SE* = 0.079, *p* < 0.001; Cohen’s *d* = 0.366). As shown in Figure 3A, at T1, the parents reported similar frequencies of the teaching of reading activities across skill levels (*F*[2, 228] = 1.56, *p* = 0.213). However, at T2 and T3, after the children entered Grade 1, the patterns began to change. Specifically, at T2, the parents of children with poor word-reading skills reported engaging in the teaching of reading more frequently than the parents of children with average word-reading skills (*M* = 0.650, *SE* = 0.193, *p* = 0.003; Hedges’ *g* = 0.593) and good word reading skills (Δ*M* = 1.641, *SE* = 0.228, *p* < 0.001; Hedges’ *g* = 1.631), and the parents of average readers engaged in more teaching of reading than the parents of good readers (Δ*M* = 0.992, *SE* = 0.199, *p* < 0.001; Hedges’ *g* = 0.895). The same pattern was observed at T3 (poor–average: Δ*M* = 0.582, *SE* = 0.189, *p* = 0.007, Hedges’ *g* = 0.526; poor–good: Δ*M* = 1.481, *SE* = 0.222, *p* < 0.001, Hedges’ *g* = 1.444; average–good: Δ*M* = 0.899, *SE* = 0.191, *p* < 0.001, Hedges’ *g* = 0.952).

When examining the patterns for each level across time, in the reading model for children with poor word reading skills, the frequency with which the parents engaged the children in the teaching of reading activities significantly increased from T1 to T2 (Δ*M* = –0.548, *SE* = 0.176, *p* = 0.010; Cohen’s *d* = 0.549) and significantly decreased from T2 to T3 (Δ*M* = 0.512, *SE* = 0.147, *p* = 0.004; Cohen’s *d* = 0.459). For children with average word reading skills, the mean level stayed the same across T1 and T2 (Δ*M* = –0.055, *SE* = 0.126, *p* = 1.000; Cohen’s *d* = 0.053) and decreased across T2 and T3 (Δ*M* = 0.455, *SE* = 0.101, *p* < 0.001; Cohen’s *d* = 0.413). For children with good word reading skills, we found a significant drop in the frequency of the teaching of reading across T1 and T2 (Δ*M* = 0.714, *SE* = 0.198, *p* = 0.002; Cohen’s *d* = 0.632) but not between T2 and T3 (*M* = 0.339, *SE* = 0.160, *p* = 0.120; Cohen’s *d* = 0.362).

In the analyses concerning children’s spelling skills (Figure 3B), we found significant main effects of Time (*F*[2, 344] = 30.08, *p* < 0.001, η_*p*_^2^ = 0.149) and Skill (*F*[2, 172] = 4.40, *p* = 0.014, η_*p*_^2^ = 0.049) The interaction of Time × Skill was not significant (*F*[4,344] = 1.52, *p* = 0.195, η_*p*_^2^ = 0.017). Thus, *post hoc* Bonferroni contrasts were calculated for only Time and Skill levels. On average, there was no mean difference in the teaching of spelling across T1 and T2, whereas the mean-level teaching of spelling at T3 was lower than the means at T1 (Δ*M* = –0.524, *SE* = 0.097, *p* < 0.001; Cohen’s *d* = 0.488) and T2 (Δ*M* = –0.659, *SE* = 0.080, *p* < 0.001; Cohen’s *d* = 0.582), the same tendencies applied to each of the three individual groups. Concerning the differences between the three groups, on average, there was no difference in the amount of teaching of spelling between the poor and average spellers or between the good and average spellers at any measurement occasion. However, the children who were good at spelling received less teaching of spelling on average than the children with poor spelling skills (Δ*M* = –0.581, *SE* = 0.196, *p* = 0.011; Hedges’ *g* = 0.638). As shown in Figure 3B, at T1, the parents reported similar frequencies of the teaching of spelling activities across skill levels (*F*[2, 228] = 0.948, *p* = 0.389). No differences were also found at T2 (*F*[2, 186] = 2.758, *p* = 0.066). However, at T3, the children with good spelling skills received less teaching of spelling from their parents compared to the children with poor spelling skills (Δ*M* = –0.880, *SE* = 0.241, *p* = 0.001; Hedges’ *g* = 0.719), and this teaching frequency of children with good spelling skills was marginally different from that of students with average spelling skills (Δ*M* = –0.478, *SE* = 0.199, *p* = 0.052; Hedges’ *g* = 0.392).

### Sensitivity Analysis

To make sure that the way we treated our parental teaching variables did not influence the results, we conducted sensitivity analyses. To this end, we ran the same models with the parental teaching variables but with the category “not anymore” forming a separate category, “not anymore” being coded as missing, the categories “not anymore” and “never” being combined (as is reported in the present study), and the category “not anymore” forming a dichotomous external control variable. In all four cases, the same trend of results was obtained, suggesting robustness of the findings irrespective of which response scale for the parental teaching variables was used.

In addition, our teaching measure included three items for reading and three items for spelling, with each item varying in difficulty, and this raises the question of whether they all captured developmentally appropriate home literacy activities. To address this, we ran another set of analyses where only the most advanced items (single item “teaching to read words” for teaching of reading and single item “teaching to spell words” for teaching of spelling) were used in our models. Once again, these analyses did not reveal any substantial differences from the results reported; thus, they confirmed the robustness of our findings.

## Discussion

The aim of the present study was to examine the longitudinal associations between the frequency of parental teaching activities (in the domains of reading and spelling) and children’s word reading and spelling skills from the end of kindergarten to the end of Grade 1. The results showed that higher scores on the word reading and spelling assessments at the end of kindergarten and the beginning of Grade 1 predicted lower frequencies of parental teaching of reading and spelling. However, the frequency of the parental teaching of reading or spelling did not predict children’s skills, with the exception of one negative association between the teaching of reading at the end of kindergarten and word reading at the beginning of Grade 1. Moreover, word reading and spelling skills were relatively stable across time, whereas the frequency of parental teaching was less stable and even non-significant during the transition from kindergarten to Grade 1. This suggests that in the first semester of Grade 1, parents already reevaluate their teaching of reading and spelling and adapt their frequency of teaching to their children’s needs for support.

### Parental Teaching of Reading and Spelling and Children’s Reading and Spelling Skills

Our first research question concerned the extent to which the frequency of the parental teaching activities would concurrently relate to and longitudinally predict the children’s word reading (RQ 1a) and spelling skills (RQ 1b). Contrary to our expectation that we would find positive associations in kindergarten, we found that the correlations between the teaching of reading and children’s word reading and the teaching of spelling and children’s spelling skills were already negative at the end of kindergarten. This was unexpected, given that previous evidence showed that the negative association was common only after the transition to Grade 1 (Silinskas et al., 2010, 2012; Sénéchal and LeFevre, 2014).

There might be a few reasons for this result. One possibility is that learning to read or spell accurately is not among the explicit goals of kindergarten education; the parents of kindergarteners are not yet supposed to monitor these skills or worry about their children’s performance in this regard. However, it seems that many parents do think that children should be able to recognize letters and sounds and be able to read and spell some basic words before entering Grade 1. Perhaps because of this belief, parents may engage in more frequent teaching before their children enter Grade 1, and this may be done especially in response to the children’s low skills in reading and spelling. It is also possible that other parental beliefs that were not assessed in the present study, such as expectations of children’s performance (Aunola et al., 2002; Martini and Sénéchal, 2012; Sénéchal and LeFevre, 2014; Silinskas et al., 2020b) or parental trust in their children’s teachers/schools (Lerkkanen et al., 2013), might have shaped our results. Therefore, more research is needed on how parental beliefs relate to parental teaching activities. Finally, it should be pointed out that although the links between teaching and skills were negative, we found positive links between maternal education and reading and spelling scores across all time points. This suggests that other aspects of family background related to parental education that are not captured by the teaching activities might account for the development of reading and spelling skills (e.g., shared genetic background, interaction quality of the literacy activities, availability of the literacy-related resources, amount of books in the home, etc.).

Contrary to both the results of previous studies and our own expectations (Sénéchal and LeFevre, 2014; Silinskas et al., 2020b), we found negative longitudinal associations between the teaching of reading in kindergarten and children’s word reading skills at the beginning of Grade 1. Previously, the negative longitudinal associations were found only in primary school—for example, across Grade 1 (Silinskas et al., 2020b) or across Grades 1 and 2 (Sénéchal and LeFevre, 2014)—whereas we found that they already existed across the transition to Grade 1. Interestingly, this pattern applied only to the domain of reading, indicating that parents may be especially sensitive to the early development of their children’s reading skills. This further suggests that, especially in the domain of reading, parental engagement in the frequent teaching of reading may not always be carried out appropriately. For instance, parents may lack the competencies to adapt their instruction to children’s skill levels or other characteristics, such as their motivation, self-regulation, or personalities. Additionally, if parental teaching activities differ from what children are exposed to in the kindergarten environment, this may confuse the children and undermine their reading acquisition.

In all other cases (domains and times), our findings did not show significant longitudinal associations between parental teaching activities and children’s subsequent literacy skills. There may be a few possible explanations for this. For instance, the effects of parental teaching activities can be time limited or evanescent. These claims have been supported by previous studies showing that the effect of home literacy teaching activities is reduced or fades away as soon as children are exposed to Grade 1 instruction in reading and spelling (Manolitsis et al., 2011; Silinskas et al., 2012). Another reason for the lack of a relationship between parental teaching and subsequent word reading and spelling skills is the specificity of our measures. We used questions to determine parental teaching behavior and tested children on skills that were appropriate for their age/stage of transition from kindergarten to Grade 1 (Lerkkanen et al., 2006–2016; Gedutiene, 2008; LR Ministry of Education, Science and Sports, 2014a). By the end of Grade 1, the range of parental teaching activities and children’s literacy skills might have become wider due to the increasing demands of the curriculum (e.g., practicing reading comprehension, spelling complicated words, and producing text). Future research needs to address this point by including a wider variety of skills, as well as teaching behaviors with different levels of difficulty.

### Effect of Children’s Skills on the Parental Teaching of Reading and Spelling

Our second research question concerned the extent to which children’s word reading skills predict the parental teaching of reading (RQ 2a), and the extent to which children’s spelling skills predict parental engagement in the teaching of spelling (RQ 2b). As expected, we found negative longitudinal paths between children’s skills and subsequent parental teaching activities across the transition from kindergarten to Grade 1 and across Grade 1. These were our main and most consistent findings in both domains (reading and spelling) across time (transition to Grade 1 and across Grade 1): The parents seemed to adjust their teaching level depending on the children’s skill levels.

The present findings extend the previous research by demonstrating that the results reported in the domain of reading (Silinskas et al., 2010, 2012; Sénéchal and LeFevre, 2014; Ciping et al., 2015) also apply to the domain of spelling. Moreover, we found that children’s skills in kindergarten already predicted increased teaching activities during the first semester of Grade 1. One previous study found these associations to be limited to the domain of reading and only later in the school career from the end of Grade 1 to Grade 2 (Sénéchal and LeFevre, 2014). In that study, children were learning to read an opaque language (i.e., English speakers were learning to read French in a French immersion program in Ontario, Canada) and were younger (4-year-old kindergarteners and 6-year-old Grade 1 students) than our participants. However, similar associations were also demonstrated in earlier studies on children entering Grade 1 (at the same age as our participants) who were learning to read with a transparent orthography. In particular, in two separate samples of children learning to read a highly transparent Finnish language (Silinskas et al., 2010, 2012), the associations became negative in Grade 1. Thus, the time at which the parental teaching of reading and spelling activities are assessed is important, and the results may be related to language transparency and the child’s developmental stage.

There are other interesting and novel aspects of our study that should be acknowledged. First, the present study clarified the results of previous work on the nature of the negative associations between reading and spelling skills and parental teaching. In particular, we clarified that it is the parents of good readers and spellers who start reporting less teaching in Grade 1 in comparison to the parents of other children who maintain similar mean levels of parental teaching. It was only in the domain of reading and only in the first semester of Grade 1 that the parents of good readers decreased their teaching frequencies, while the parents of poor readers tended to increase theirs. Another interesting result concerned the difference in the strength of the parents’ responsiveness in the reading and spelling models. In particular, in the spelling model, the strength of the longitudinal paths between spelling skills and parental teaching of spelling was somewhat similar across time (–0.369 and –0.320). However, for the reading model, the cross-lagged longitudinal paths from reading fluency to parental teaching of reading were substantially different. In particular, the parents were more reactive with their teaching of reading at the beginning of Grade 1 (–0.622), in comparison to their responsiveness to children’s skill levels at the end of Grade 1 (–0.266). This highlights the importance of domain specificity. Taken together, these results suggest that parents are particularly responsive to their children’s skills in reading, and especially so at the beginning of formal schooling in Grade 1. This is understandable, given that the development of reading skills is prioritized at the very beginning of formal schooling in Grade 1; accuracy in spelling skills may be prioritized later and may need more time to develop (Alcock and Ngorosho, 2003; Leppänen et al., 2006; Landerl and Wimmer, 2008; Hirvonen et al., 2010; Georgiou et al., 2020). Overall, it was determined that the parents of children with good reading and spelling skills tend to provide less teaching at home, and it is only in the domain of reading that parents of low-performing children may provide more teaching than those of other children.

Theoretically, our findings support the transactional theories of child socialization (Sameroff, 2010) and especially theories of the evocative effect of children’s characteristics on parental behaviors (Scarr and McCartney, 1983; Nurmi, 2012). For instance, it has been suggested that parental involvement in children’s academic development can be explicitly and implicitly initiated by children (Hoover-Dempsey and Sandler, 1995; Green et al., 2007). In other words, if parents perceive that their children explicitly invite them to become involved (e.g., by actively asking to read or write together), parents become involved in these academic-related activities at home more often (Green et al., 2007). Apart from explicit invitations from children, parents may be responsive to their children’s implicit invitations in the form of certain characteristics (e.g., low performance, low motivation to learn reading and spelling, or distracted behavior in learning situations) and may adapt the frequency of reading and spelling activities at home accordingly (Silinskas et al., 2015). Therefore, the results of our study highlight the importance of implicit child invitations that often have an evocative impact on parental responses in academic contexts (Scarr and McCartney, 1983; Rutter, 1997; Nurmi, 2012).

### Limitations

Some limitations of the present study need to be acknowledged. First, this study suggests a direction of longitudinal associations but cannot make causal claims about the relation of influence. Although we gathered longitudinal data every 6 months (from the end of kindergarten to the end of Grade 1), experimental and intervention studies are needed to support causal claims. Second, parental self-reports were used to assess the frequency of the parental teaching of reading and spelling. Measuring the frequency of these teaching activities may not capture the richness or quality of the interactions between parents and children (Aram and Levin, 2002; Levin and Aram, 2005; Aram et al., 2013; Sénéchal et al., 2017). Therefore, future studies would benefit from the use of observations, audio/video recordings of parent–child interactions, and/or experience-sampling approaches (i.e., intensive data gathering or diary data). They would also benefit from the assessment of a wider range of teaching activities (learning to read sentences, understanding read texts, creative writing, etc.). Third, our sample consisted of somewhat high-SES families. It was also relatively homogeneous in terms of cultural and ethnic backgrounds. Although in terms of the home languages, the sample was relatively representative of the Lithuanian population, the inclusion of high-SES families limits the generalizability of our results. Therefore, future studies should try to recruit and maintain the interest of families with different SES backgrounds to participate in longitudinal studies. Fourth, although other studies have found that children’s intelligence can be an important correlate of literacy skills (Niklas and Schneider, 2015), unfortunately, the present study did not collect information to support this determination. Future studies, however, should use information about a child’s intelligence to control for its effects on children’s literacy development and parental involvement. Finally, the study presents evidence from a new cultural environment, Lithuania (the northeastern part of Europe), where children enter Grade 1 at the age of seven and are learning to read and spell with a relatively transparent orthography. In this way, our results can be generalizable to other countries where first graders start learning to read and spell with similar orthographies (e.g., Finnish or Greek). Learning to read and spell in opaque orthographies may require more time (Seymour et al., 2003), and thus the results described here may appear later than Grade 1 (Sénéchal and LeFevre, 2014). These assumptions remain to be tested empirically in future research.

## Conclusion

Our findings extend the previous research by showing that parents adapt the frequency of their teaching activities to their children’s reading and spelling skill levels. In particular, the parents of good word readers and spellers decrease their teaching frequencies of reading and spelling across time. Particular attention should be paid to the domain of reading at the very start of Grade 1, as the parents of poor readers also increase their teaching frequencies of reading over time and in comparison to the parents of children with average and good word reading skills. Taken together, our findings emphasize the importance of children’s developmental stages and the crucial role of school transition, after which the frequency of parental home teaching activities is adapted to the needs of the children.

Our study expands the previous literature in a few ways. First, it reports longitudinal evidence, wherein parental reports of the frequency of teaching were obtained three times every 6 months from the end of kindergarten to the end of Grade 1. Longitudinal studies, especially across the transition to Grade 1, remain rare. Second, we ran separate models for reading and spelling. While the teaching of reading and its relationship to reading skills is relatively well understood, the frequency of the teaching of spelling and its relationship to spelling skills remains under-researched. Third, the findings were based on a large sample size and were obtained by applying sophisticated statistical techniques (e.g., RI-CLPM) that controlled for the between-subject differences and allowed for the observation of within-person change. None of the previous studies on home literacy used this approach.

From a practical standpoint, the findings of the current study should not be taken as evidence of parents’ inability to effectively involve themselves in their children’s learning. Rather, the results emphasize the importance of parental teaching activities in kindergarten and Grade 1. In particular, parental awareness and responsiveness to children’s performance in reading and spelling is a useful first step. The next step should be to make use of this parental time and effort in the best way possible. In Grade 1, teachers start to provide more explicit feedback to parents about their children’s progress in reading and spelling through report cards and parent–teacher meetings. This explicit feedback has been shown to be especially powerful in predicting parental engagement in the academic domain (Green et al., 2007). Therefore, to get parents involved in the most optimal way, teachers and educators should communicate the content and instructional goals of the lessons and discuss the concrete ways in which parents can contribute to their children’s learning processes. In other words, emphasis should be placed not only on the frequency but also on the ways in which instructional goals can be met in collaboration between parents, teachers, and other education professionals.

## Data Availability Statement

The raw data supporting the conclusions of this article will be made available to any qualified researcher, if requested from the first author.

## Ethics Statement

The studies involving human participants were reviewed and approved by the Ethical Committee of the University of Jyväskylä on 3.5.2017. Written informed consent to participate in this study was provided by the participants’ legal guardian/next of kin.

## Author Contributions

GS made a substantial contribution to the conception and drafting of the manuscript. KA made a substantial contribution to analyses and revisions. M-KL made a substantial contribution to the conception and revisions. SR made a substantial contribution to data collection. All authors contributed to the article and approved the submitted version.

## Conflict of Interest

The authors declare that the research was conducted in the absence of any commercial or financial relationships that could be construed as a potential conflict of interest.
